# Scissor–CIBERSORTx Deconvolution Reveals Functional Heterogeneity of CTAL/aTAL Cells and Associated Biomarkers in Renal Fibrosis

**DOI:** 10.3390/cimb48020215

**Published:** 2026-02-16

**Authors:** Hengping Wang, Yuan Zhang, Jiale Li, Ying Fu, Huiyan Wang

**Affiliations:** Jilin Collaborative Innovation Center for Antibody Engineering, Jilin Medical University, Jilin 132013, China; wanghengping0116@163.com (H.W.); zy970415@sina.com (Y.Z.); 18243123653@163.com (J.L.)

**Keywords:** renal fibrosis, single-cell RNA sequencing analysis, adaptive thick ascending limb cell, cortical thick ascending limb cell, scissor, CIBERSORTx

## Abstract

Renal fibrosis (RF) represents a major pathological outcome of chronic kidney disease, currently accompanied by extremely limited therapeutic strategies. To decipher key cellular and molecular drivers, we integrated single-cell and bulk transcriptomic profiles for comprehensive analysis. Based on the RF-related single-cell and bulk transcriptomic data, key cell subtypes were identified through Scissor analysis, custom signature matrix construction via CIBERSORTx, and Weighted Gene Co-Expression Network Analysis (WGCNA). Subsequently, key subtype-related biomarkers were identified through the expression analysis, and functional enrichment analysis for biomarkers was conducted to elucidate the potential mechanisms by which biomarkers regulate RF. Through comprehensive profiling, thick ascending limb (TAL) cells were predominant and displayed marked heterogeneity in renal fibrosis (RF), with cortical TAL (CTAL) and adaptive TAL (aTAL) identified as principal subtypes. A set of candidate biomarkers was identified. Quantitative polymerase chain reaction (qPCR) validation in mouse models confirmed aberrant expression of these biomarkers, with STAT1 and PARP8 upregulated and HS6ST2, PTGER3, and TMEM207 downregulated in RF. Furthermore, functional enrichment analyses indicated that these biomarkers were associated with pathways underlying metabolic reprogramming and immune perturbation. Our study implicates CTAL and aTAL as central cellular players in RF and identifies their associated biomarkers. These experimentally validated biomarkers provide novel targets and repurposing opportunities for RF therapeutic intervention.

## 1. Introduction

Chronic kidney disease (CKD) is defined as abnormalities in kidney structure or function persisting for more than three months, with significant morbidity and mortality [1]. Renal fibrosis (RF) represents a key pathological feature and common endpoint of CKD [2], which can be initiated by various factors, including infections, ischemia, diabetes, autoimmune diseases, urinary tract obstruction, and toxic exposures [3,4]. Characteristic morphological changes in RF include glomerulosclerosis, tubular atrophy, chronic interstitial inflammation, fibrosis, and capillary rarefaction [1]. As RF progresses, the gradual loss of functional nephrons ultimately leads to end-stage renal disease [3]. Early detection of RF is therefore critical for timely intervention and improved patient prognosis. Currently, diagnosis of RF relies primarily on renal biopsy—an invasive procedure associated with risks such as bleeding [5]. Given this, exploring novel anti-fibrotic targets and therapeutic strategies represents a critical direction in current research.

**Figure 1:**
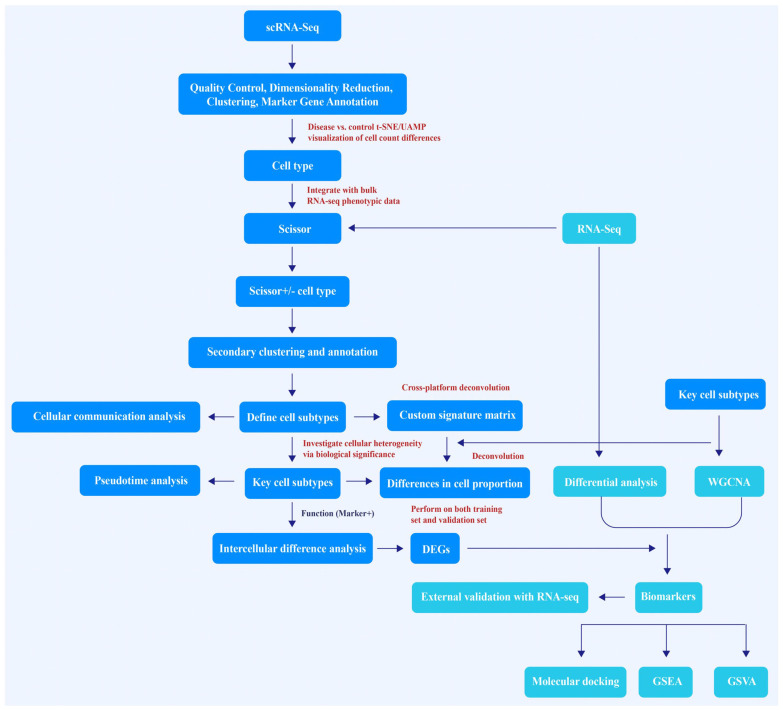
Schematic diagram of the workflow in this study. scRNA-seq, single-cell RNA sequencing; t-SNE, t-distributed Stochastic Neighbor Embedding; UMAP, Uniform Manifold Approximation and Projection; Scissor, Single-cell identification of subsets of interest via enrichment of regulators; DEGs, differentially expressed genes; WGCNA, Weighted Gene Co-expression Network Analysis; GSEA, Gene Set Enrichment Analysis; GSVA, Gene Set Variation Analysis. Blue boxes represent the primary analytical workflows derived from scRNA-seq data, while teal boxes represent the secondary validation and functional analyses that utilize bulk RNA-seq data.

Single-cell RNA sequencing (scRNA-seq) can reveal the heterogeneity of cells in tissues and the mechanism of disease [6,7], providing a new perspective for analyzing cell dynamics and interactions during RF. However, despite its advantages over bulk RNA-seq in capturing cellular diversity within complex tissues, there are several limitations, including limited sample sizes, tissue dissociation bias, and insufficient sensitivity for detecting low-expression genes [8]. To overcome the limitations of the aforementioned methods, this study integrates CIBERSORTx with the Scissor algorithm to enable collaborative analysis of single-cell and batch transcriptomic data. Specifically, the Scissor algorithm correlates single-cell transcriptomes with clinical phenotypes to identify key cell subpopulations associated with disease states [9]. Meanwhile, CIBERSORTx constructs tissue-specific feature matrices from single-cell data and employs deconvolution analysis to resolve cell type composition and proportions from batch transcriptomic data. This combined approach systematically reveals phenotypically relevant cell subpopulations and their molecular characteristics during RF progression [7,10,11]. Through this integrated analytical framework, key cellular subtypes and biomarkers implicated in RF progression were systematically delineated, thereby providing a conceptual framework and putative molecular targets for early diagnosis and precision therapeutic intervention. Furthermore, bioinformatically predicted biomarkers were validated by quantitative polymerase chain reaction (qPCR) in mouse models of renal injury and fibrosis, confirming their expression patterns and reinforcing their translational relevance. The study workflow is presented in Figure 1.

## 2. Materials and Methods

### 2.1. Data Collection

This study employed publicly available single-cell and transcriptomic datasets retrieved from the Gene Expression Omnibus (GEO) database (http://www.ncbi.nlm.nih.gov/geo/) (accessed on 21 May 2025). The secondary analysis of these pre-existing datasets was conducted in accordance with international ethical guidelines governing the reuse of anonymized genomic data, as stipulated by the ICMJE.

Through the GEO database, the GSE195718 (platform: GPL24676), GSE76882 (platform: GPL13158), and GSE135327 (platform: GPL21290/GPL11154) datasets were acquired [12,13,14]. The scRNA-seq dataset GSE195718 includes a total of 9 kidney allograft biopsies: 6 obtained from patients with chronic allograft dysfunction (CAD) accompanied by interstitial fibrosis and tubular atrophy (IFTA), and 3 from patients with stable graft function and normal or nonspecific histopathological findings, which served as controls. The bulk-RNA sequencing datasets GSE76882 (the training set) and GSE135327 (the validation set) contain 99 control and 135 RF samples, and 12 control and 18 RF samples, respectively.

### 2.2. Cell Annotation

The quality control was implemented in the Seurat (v5.1.0) package on the GSE195718 dataset [15]. Data were filtered out under the following criteria: genes were detected in <200 cells; cells with nFeature_RNA ≥ 7000; cells with nCount_RNA ≥ 30,000; and mitochondrial content (percent.mt) ≥ 10%. Followed by logarithmic normalization, the top 2000 highly variable genes (HVGs) were identified via vst method for principal component analysis (PCA). Before PCA, the ScaleData function was applied to scale the data. Based on the results of the scree plot and the PCA permutation test, the appropriate principal components (PCs) were selected for subsequent clustering analysis. Cell clustering was conducted by applying the FindNeighbors and FindClusters functions (resolution = 0.3), followed by t-SNE-based visualization. Cell types were identified by comparing the expression of marker genes obtained from previous studies among cell clusters [12,16,17,18,19,20]. Furthermore, the DoubletFinder (v2.0.4) package was employed to detect and remove doublets, and the clustering situation of various types of cells and their proportion in the samples was further examined [21].

### 2.3. Scissor Analysis

Scissor is a novel approach that utilizes the phenotypes, such as disease stage, tumor metastasis, treatment response, and survival outcomes, collected from bulk assays to identify the most highly phenotype-associated cell subpopulations from single-cell data. The workflow of Scissor is shown in Figure S1A [22]. The Scissor (v2.0.0) package was utilized to conduct association analysis in GSE195718 and GSE76882 datasets, to identify RF-associated cell types (alpha = 0.001 and family = binomial) [9]. Scissor+ denotes a positive association with the disease, Scissor− denotes a negative association, and a value of 0 indicates no statistically significant correlation. Building upon these identified RF-associated cell types, the ROGUE (v1.0) package was applied to compute heterogeneity scores for these cells [23]. Lower scores reflect greater cellular heterogeneity. Further integrating the Scissor analysis results, we selected cell types with lower heterogeneity scores and performed secondary clustering analysis on them using the same analytical procedure [24,25]. For single-cell subclusters that could not be annotated to previously reported cells, the FindMarkers function was employed to identify differentially expressed genes (DEGs) with thresholds of |log_2_FoldChange (FC)| > 0.5 and adjusted *p*-value < 0.05 between the non-annotated subclusters and other subclusters. Subsequent functional annotation was supported by Gene Ontology (GO) and Kyoto Encyclopedia of Genes and Genomes (KEGG) enrichment analyses performed on the identified DEGs. The proportion of each cell subtype within the sample was quantified and presented.

### 2.4. CIBERSORTx Analysis

CIBERSORTx is an analytical tool developed by Newman et al. to impute gene expression profiles and provide an estimation of the abundances of member cell types in a mixed cell population, using gene expression data [10]. A typical CIBERSORTx workflow involves a serial approach (Figure S1B), in which molecular profiles of cell subsets are first obtained from a small collection of tissue samples and then repeatedly used to perform systematic analyses of cellular abundance and gene expression signatures from bulk tissue transcriptomes. This process involves: transcriptome profiling of single cells or sorted cell subpopulations to define a “signature matrix” consisting of barcode genes that can discriminate each cell subset of interest in a given tissue type; applying the signature matrix to bulk tissue RNA profiles in order to infer cell type proportions and representative cell type expression signatures; and purifying multiple transcriptomes for each cell type from a cohort of related tissue samples.

In the GSE195718 dataset, the number of cells for each type was randomly downsampled through proportional sampling. Specifically, for cell types with more than 1000 cells, a maximum of 1000 cells were retained; and among these cells, 70% of the cells were resampled to meet the input requirements of the CIBERSORTx tool [10]. Subsequently, the Create Signature Matrix function was applied to generate deconvoluted single-cell data for constructing a custom signature matrix. Afterthat, utilizing the custom signature matrix, CIBERSORTx was applied to deconvolve bulk expression profiles from datasets GSE76882 and GSE135327. The inferred cell type proportions were then compared between the RF and control groups (*p* < 0.05).

### 2.5. Weighted Correlation Network Analysis (WGCNA)

WGCNA can be used for finding clusters (modules) of highly correlated genes, for summarizing such clusters using the module eigengene or an intramodular hub gene, for relating modules to one another and to external sample traits (using eigengene network methodology), and for calculating module membership measures. Correlation networks facilitate network-based gene screening methods that can be used to identify candidate biomarkers or therapeutic targets [26]. The workflow of WGCNA is shown in Figure S1C. WGCNA was performed via the WGCNA (v1.71) package on the GSE76882 dataset to cluster samples and identify potential outliers for possible exclusion [27]. The soft threshold was selected to maximize the conformity of gene interactions to a scale-free distribution and to ensure that the mean connectivity tends toward zero (R^2^ > 0.85). According to the standard of the dynamic tree cutting algorithm, the minimum module size was set to 200 genes to guide the cutting and merging of gene modules. The cell subtypes exhibiting significant differences in proportion between the RF and control groups were treated as traits to calculate correlations with gene co-expression modules via the Pearson algorithm. The module exhibiting the highest correlation with traits was selected as the key module. Genes in the key module were key module genes. Cell subtypes showing both the strongest correlations with the key module and statistically significant differences in proportion between groups in the GSE76882 and GSE135327 datasets, along with consistent trends, were identified as key cell subtypes.

### 2.6. Identification of Biomarkers Related to RF Progression in Key Cell Subtypes

Differential expression analysis was implemented using the limma (v3.52.4) package to identify notable DEGs between RF and control samples in the GSE76882 dataset (|log_2_FC| > 1 and adjusted *p* < 0.05) [28]. Meanwhile, within the custom signature matrix, the FindMarkers function was applied to perform differential expression analysis of key cell subtypes in comparison to all other subtypes, aiming to identify DEGs specific to these cell types. For each key cell type, the corresponding DEGs were intersected with both the DEGs between the RF and control groups and the key module genes. Subsequently, expression levels of these intersected gene sets were analyzed in the GSE76882 and GSE135327 datasets. Genes exhibiting significant differential expression between the RF and the control groups, with consistent expression trends across both datasets, were designated as biomarkers. Expression levels of these were visualized in the GSE195718 dataset.

### 2.7. Cell Communication and Pseudotime Trajectory Analyses

Pseudotime analysis is a computational approach used to infer the dynamic, continuous developmental or transition trajectory of cells based on high-throughput sequencing data. It simulates a “virtual timeline” (pseudotime) that reflects the sequential changes in cells from an initial state to a terminal state, without relying on actual temporal sampling [29]. This method can identify the ordered progression of cellular states, key genes driving the transition, and the dynamic expression patterns of genes along the trajectory, which is particularly useful for exploring the dynamic process of renal fibrosis and cell fate determination in our study. The CellChat package (v1.6.1) was employed to conduct intercellular communication analysis in the GSE195718 dataset, with particular emphasis on key cell subtypes. Additionally, pseudotime trajectory analysis was conducted adopting the monocle package (v2.26.0) on cell types to further investigate the functional dynamics of different cell types or states during RF progression [30]. Simultaneously, the expression of biomarkers during the pseudotime process was demonstrated.

### 2.8. Functional Enrichment Analysis

To investigate how varying biomarker expression levels influence pathway dynamics during RF progression, the clusterProfiler package (v4.7.1.001) was utilized for Gene Set Enrichment Analysis (GSEA) [31]. The gene-biomarker correlations in the GSE76882 dataset were computed, followed by gene ranking by correlation coefficient for GSEA (|NES| > 1 and adjusted *p* < 0.05). To further characterize pathway-level changes, Gene Set Variation Analysis (GSVA) was implemented. The GSVA package was utilized to quantify pathway activity scores in the GSE76882 dataset, and the limma package was employed to conduct differential pathway analysis between the RF and control groups (|t| > 2 and *p* < 0.05). All analyses adopted the KEGG gene set (c2.cp.kegg.v7.0.symbols.gmt) from MSigDB (https://www.gsea-msigdb.org/gsea/msigdb) (accessed on 4 June 2025) as a reference set.

### 2.9. Animal Model

To validate bioinformatics findings related to renal injury and fibrosis, two well-established mouse models were employed in this study: the folic acid (FA)-induced acute kidney injury model and the unilateral ureteral obstruction (UUO)-induced chronic renal fibrosis model. These models were used to quantify the expression and temporal dynamics of five predicted biomarkers. Male C57BL/6 mice (6 weeks old, weighing 20–25 g) were purchased from Liaoning Changsheng Biotechnology Co., Ltd. (Shenyang, China). All animals were housed in a specific pathogen-free (SPF) facility and randomly assigned into three groups: a wild-type normal control group (WT-normal group), a folic acid-induced acute kidney injury group (FA group), and a unilateral ureteral obstruction-induced chronic renal fibrosis group (UUO group). Mice in the FA group received a single intraperitoneal injection of folic acid (250 mg/kg), and samples were collected 48 h later to establish the acute kidney injury model. Mice in the UUO group underwent unilateral ureteral ligation, and tissues were harvested after 14 days to induce chronic renal fibrosis and injury.

All animal experiments were conducted in strict accordance with ethical guidelines and were approved by the Animal Ethics Committee of Jilin Medical University (Approval No. JLYY-ETH-2024-007).

### 2.10. qPCR

Total RNA was extracted from each sample using TRIzol reagent (Thermo Fisher Scientific, Carlsbad, CA, USA). Approximately 2000 ng of total RNA was reverse-transcribed into complementary DNA (cDNA) using the PrimeScript RT reagent kit (Takara Bio, Kusatsu, Japan). mRNA expression levels were quantified by real-time quantitative PCR with SYBR Green fluorescence dye on an applied biosystems qPCR system. All primers used in this study were synthesized by Sangon Biotech (Shanghai, China). The expression levels of target mRNAs were normalized to the internal control gene encoding ribosomal protein small subunit 16 (Rps16) for subsequent comparative analysis. Detailed primer sequences are listed in Table 1.

### 2.11. Histological Analysis

For morphological evaluation, renal tissues from normal mice, FA-induced acute kidney injury mice, and UUO-induced chronic kidney fibrosis mice (14 days post-operation) were paraffin-embedded and sectioned at a thickness of 5 μm. Subsequently, the sections were deparaffinized, rehydrated, and subjected to sequential staining with hematoxylin and eosin (H&E) and periodic acid–Schiff (PAS).

### 2.12. Drug Prediction and Molecular Docking

Potential therapeutic drug compounds targeting the biomarkers were predicted utilizing the DSigDB (https://dsigdb.tanlab.org/) (accessed on 9 June 2025). Molecular docking was then conducted to assess the binding affinity between these biomarkers and their candidate drugs. Drug structures were selected based on their significance (*p*-value). Protein structures for the biomarkers were obtained from UniProt (https://www.uniprot.org/) (accessed on 9 June 2025), while 3D compound structures were sourced from PubChem (https://pubchem.ncbi.nlm.nih.gov/) (accessed on 9 June 2025). Water molecules and small-molecule ligands were removed through AutoDock Vina (v1.2.3) software, followed by molecular docking analysis conducted employing AutoDock Vina [32]. Ligand–receptor binding energies were computed, with values ≤ −5.0 kcal/mol defined as indicative of favorable binding activity.

### 2.13. Statistical Analysis

R software (https://www.r-project.org/) was utilized for statistical analyses. Differences between groups were assessed utilizing the Wilcoxon test, with a *p*-value of <0.05. Statistical analysis for qPCR was performed using GraphPad Prism 8.0 software (GraphPad Software, San Diego, CA, USA). Differences among experimental groups were compared by one-way analysis of variance (ANOVA). Data are presented as the mean ± standard error of the mean (SEM). A *p*-value < 0.05 was considered statistically significant.

## 3. Results

### 3.1. Single-Cell Atlas Reveal RF-Associated Renal Cell Heterogeneity and Functional Differentiation of Thick Ascending Limb (TAL) Cells

**Figure 2:**
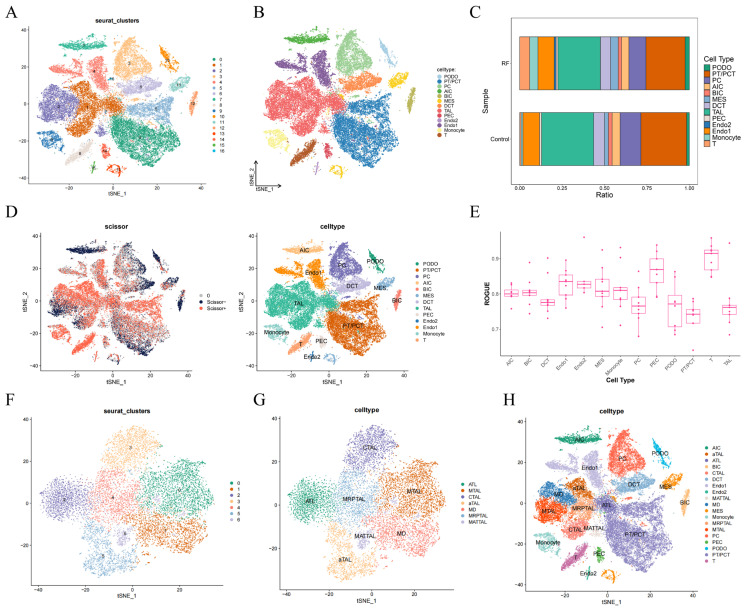
Identification of cell subtypes. (**A**) t-SNE clustering plot of 17 clusters in the GSE195718 dataset; (**B**) t-SNE plot illustrating the distribution of 13 cell types; (**C**) the cellular composition of each cell type in renal fibrosis (RF) and control groups; (**D**) t-SNE plots showing the correlation between cell types and RF; (**E**) The ROGUE scores for each cell type; (**F**) t-SNE plot illustrating the distribution of 7 subclusters of thick ascending limb (TAL); (**G**) t-SNE plot illustrating the distribution of 7 cell subtypes of TAL; (**H**) t-SNE plot illustrating the distribution of all cell types; Scissor+: positive correlation; Scissor−: negative correlation; and 0: no significant correlation.

Following data filtering, 47,387 cells, comprising 27,263 cells from RF samples and 20,124 cells from control, as well as 30,767 genes, were retained (Figure S1A). PCA was performed on the top 2000 HVGs, and the top 30 PCs were selected for subsequent analysis (Figure S2B–D). Through clustering analysis of cell clusters, a total of 17 categories were identified. Subsequently, cell type annotation was conducted on the 17 clusters derived from the clustering analysis, resulting in the identification of 13 distinct cell types, namely podocytes (PODO), proximal tubule (PT)/proximal convoluted tubule (PCT) cells, princal cells (PC), type A intercalated cells (AIC), type B intercalated cells (BIC), mesangial cells (MES), distal convoluted tubule cells (DCT), TAL, parietal epithelial cells (PEC), type 2 endothelial cells (Endo2), type 1 endothelial cells (Endo1), monocytes, and T cells (Figure 2A,B and Figure S2E). Following the removal of 3554 high-confidence doublets (7.5% of the dataset), cell boundaries became more distinct with significantly reduced impact of technical noise on biological signals (Figure S2F). Among these cell types, the proportions of TAL and PT/PCT in the samples were both relatively high (Figure 2C). The Scissor algorithm demonstrated that a substantial proportion of cells in PT/PCT, TAL, Endo, Monocytes, and T populations exhibited positive associations with RF. Notably, a considerable number of cells within the PT/PCT, TAL, and AIC also displayed negative correlations with RF (Figure 2D). These findings suggest that these cell types might exhibit a certain degree of heterogeneity. This hypothesis was further supported by the ROGUE analysis, which revealed relatively lower scores for PT/PCT, TAL, and AIC (Figure 2E). Based on the integrated analysis of overall cell proportion, heterogeneity, and disease-related correlations, TAL was selected for secondary clustering. Among the seven cell subclusters, cluster 0 was annotated as medullary TAL (MTAL), cluster 1 as macula densa (MD), cluster 2 as ascending thin limb cell (ATL), cluster 3 as cortical TAL (CTAL), and cluster 5 as adaptive TAL (aTAL). Cluster 4 was involved in protein-targeted transport and specific subcellular structure localization and was named metabolically reprogrammed progenitor TAL (MRPTAL); cluster 6 was enriched in amino acid metabolism, as well as membrane transport-related biological processes, and was named metabolically adapted transport TAL (MATTAL) (Figure 2F,G, and Figure S2G, Table S1). As shown in Figure 2H, the annotation cell types and subtypes were summarized.

### 3.2. CTAL and aTAL Were Identified as the Key Cell Subtypes

**Figure 3:**
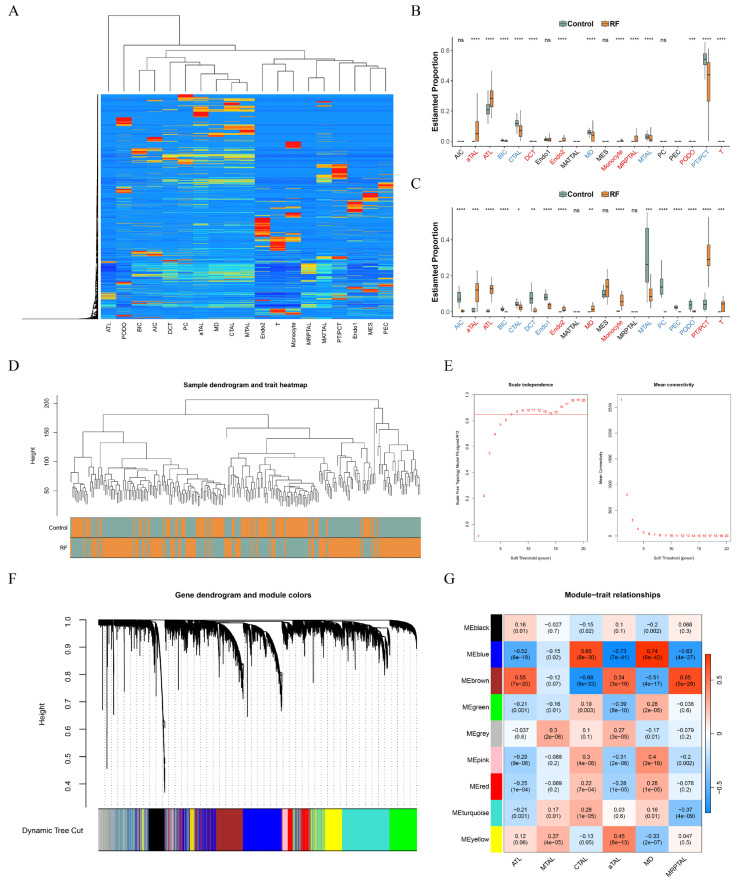
Identification of key cell subtypes from TAL. (**A**) Heatmap of CIBERSORTx deconvolution results. (**B**) Box plot showing the proportion of cells in the RF group and the control group in GSE76882 dataset; (**C**) box plot showing the proportion of cells in the RF group and the control group in GSE135327 dataset; (**D**) clustering results of WGCNA in GSE76882 dataset; (**E**) selection of soft threshold; (**F**) gene dendrogram and module colors; and (**G**) heat map of the correlation between modules and traits. ns: not significant, * *p* < 0.05, ** *p* < 0.01, *** *p* < 0.001, and **** *p* < 0.0001.

Through the CIBERSORTx tool, a unique custom signature matrix was constructed (Supplementary Table S2). After deconvolution, we observed that the proportions of CTAL and MTAL (downregulated), as well as aTAL and ATL (upregulated), showed significant differences between groups in both GSE76882 and GSE135327 datasets (Figure 3A–C). In the subsequent WGCNA, clustering results showed that sample grouping was consistent, indicating no need to exclude any samples (Figure 3D). Using a soft threshold of eight, nine distinct modules were identified (Figure 3E,F). The brown (1775 genes) and blue (2924 genes) modules had strong correlations with the proportions of TAL cell subtypes, except for MTAL, and were therefore identified as key modules, containing 4699 key module genes (Figure 3G). CTAL and aTAL displayed strong correlations with these key modules (|cor| > 0.53). Because of the significant differences between groups, they were classified as key cell subtypes.

### 3.3. Cell Communication and Pseudotime Analysis of TAL Subtypes

**Figure 4:**
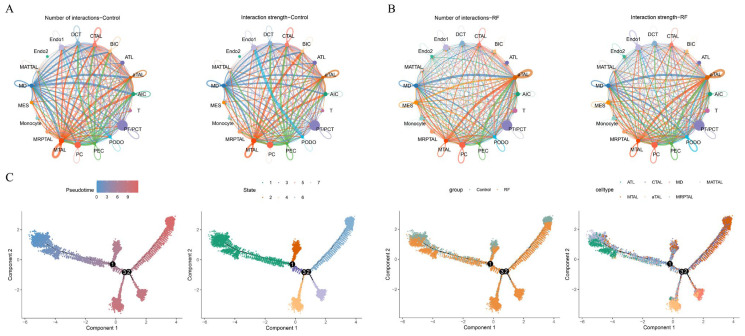
Cellular communication and pseudotime analysis. (**A**) Cellular communication network illustrating the number (left) and strength (right) of interactions among cell types in the control group; (**B**) Cellular communication network illustrating the number (left) and strength (right) of interactions among cell types in the RF group; (**C**) Time trajectory of TAL in pseudotime trajectory analysis (upper left); Cell state distribution of TAL in pseudotime trajectory analysis (upper right); Time trajectory of TAL in the RF group and the control group (lower left); Cell subtypes distribution of TAL in pseudotime trajectory analysis (lower right). Nodes 1–3 indicate trajectory branch points (bifurcations), highlighting positions of cell fate divergence during differentiation.

Cell communication analysis revealed that the number and intensity of interactions involving aTAL did not show significant differences between the RF and control groups. In contrast, CTAL exhibited higher interaction counts and intensities in the control group compared to the RF group (Figure 4A,B). Moreover, given the critical role of TAL and its subtypes in RF, pseudotime analysis was performed to explore their dynamic changes during the progression. TAL differentiates into six stages during the pseudotime development. CTAL was predominantly found in the early stage (stage 1), and aTAL mainly appeared in the middle stage (stage 4) (Figure 4C).

### 3.4. STAT1, PARP8, HS6ST2, PTGER3, and TMEM207 Were Biomarkers for RF

**Figure 5:**
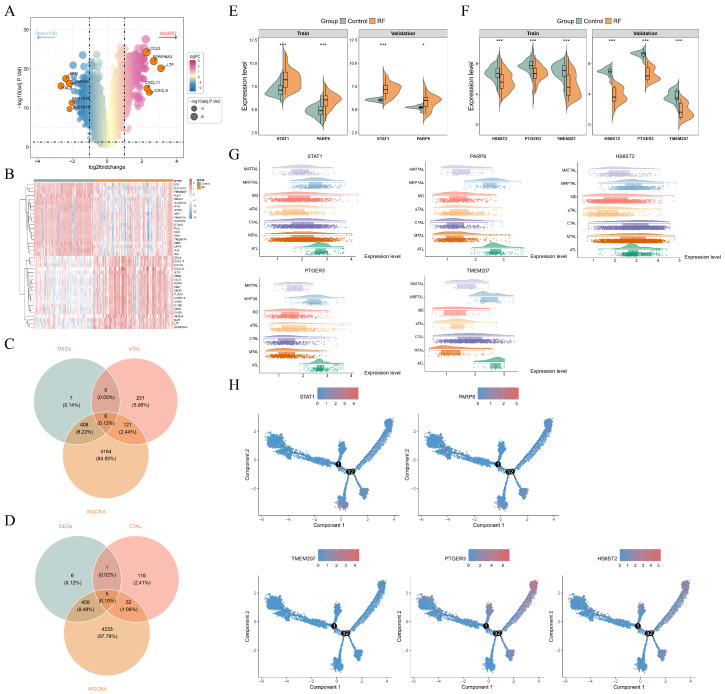
Identification of biomarkers. (**A**) Volcano plot of differentially expressed genes (DEGs) between the RF group and the control group in the GSE76882 dataset; (**B**) heatmap of top 20 upregulated and downregulated DEGs in the GSE76882 dataset, ranked by |log_2_FC|; (**C**) Venn diagram of 378 DEGs in aTAL, 421 DEGs in the GSE76882 dataset, and 4699 key module genes; (**D**) Venn diagram of 174 DEGs in cTAL, 421 DEGs in the GSE76882 dataset, and 4699 key module genes; (**E**) violin plot of STAT1 and PARP8 expression across samples in the GSE76882 and GSE135327 datasets; (**F**) violin plot of HS6ST2, PTGER3, and TMEM207 expression across samples in the GSE76882 and GSE135327 datasets; (**G**) expression of biomarkers in cell subtypes of TAL; and (**H**) biomarker expression in pseudotime trajectory analysis. Nodes 1–3 indicate trajectory branch points (bifurcations), highlighting positions of cell fate divergence during differentiation. * *p* < 0.05 and *** *p* < 0.001.

In the GSE76882 dataset, there were 421 DEGs, including 158 downregulated and 263 upregulated genes in RF samples (Figure 5A). The heatmap separately showed the expression patterns of the top 20 upregulated and downregulated DEGs (Figure 5B). There were 378 DEGs between aTAL and other TAL subgroups, while CTAL had 174 DEGs. Thereafter, 378 DEGs in aTAL, 421 DEGs in the GSE76882 dataset, and 4699 key module genes were intersected, resulting in the 6 intersected genes (Figure 5C). For CTAL, there were five intersected genes (Figure 5D). STAT1 and PARP8 in aTAL were significantly upregulated in the RF group across GSE76882 and GSE135327 datasets, while HS6ST2, PTGER3, and TMEM207 in CTAL were significantly downregulated (Figure 5E,F). Therefore, these five genes served as potential biomarkers. The expression of all biomarkers in TAL cell subtypes was displayed in Figure 5G. During the differentiation of TAL cells, it was worth noting that the expressions of PTGER3 and HS6ST2 increased at the terminal stage of differentiation (Figure 5H).

### 3.5. The Biomarkers Were Enriched in Metabolism and Immune Dysregulation Pathways

**Figure 6:**
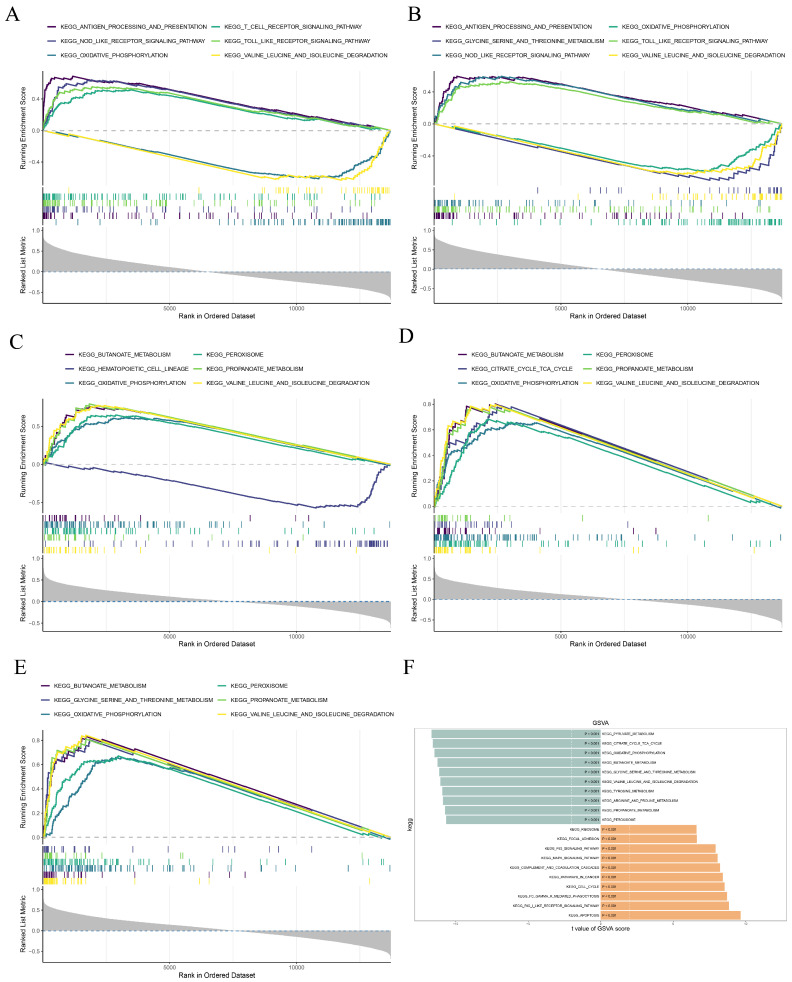
Enrichment analysis of biomarkers. (**A**) Gene Set Enrichment Analysis (GSEA) of STAT1; (**B**) GSEA of PARP8; (**C**) GSEA of HS6ST2; (**D**) GSEA of PTGER3; (**E**) GSEA of TMEM207. The GSEA enrichment analysis graph is divided into two parts: the enrichment score line section, where the x-axis represents the sorted genes, and the y-axis represents the corresponding Running ES. There is a peak in the line graph, which is the enrichment score of this pathway gene set. The genes before the peak are the core genes of this gene set; the lines in the lower part mark the genes under this gene set. (**F**) Gene Set Variation Analysis (GSVA) between the RF and control groups in the GSE76882 dataset, green represents down-regulated pathways, and orange represents up-regulated pathways.

The function of the biomarker was analyzed, and the results showed STAT1, PARP8, HS6ST2, PTGER3, and TMEM207 were enriched in 73, 69, 67, 68, and 74 pathways, respectively (Figure 6A–E, Table S3). Their co-enrichment in energy metabolism pathways (e.g., oxidative phosphorylation and butanoate/propanoate metabolism) and immune dysregulation pathways (e.g., graft-versus-host disease and systemic lupus erythematosus) suggested dual roles in metabolic homeostasis and inflammatory injury responses. Notably, GSVA identified 155 pathways markedly different between the RF and control groups, including the aforementioned ones (Figure 6F, Table S4).

### 3.6. Experimental Validation of Elevated Expression of Hub Biomarkers in a Murine Model of Renal Fibrosis

To evaluate renal pathological changes under normal, acute injury, and fibrotic conditions, histological analyses were performed on kidney tissues from three groups—normal control (normal), folic acid-induced acute kidney injury (FA-2 d), and unilateral ureteral obstruction-induced chronic fibrosis (UUO-14 d)—using H&E and PAS staining.

**Figure 7:**
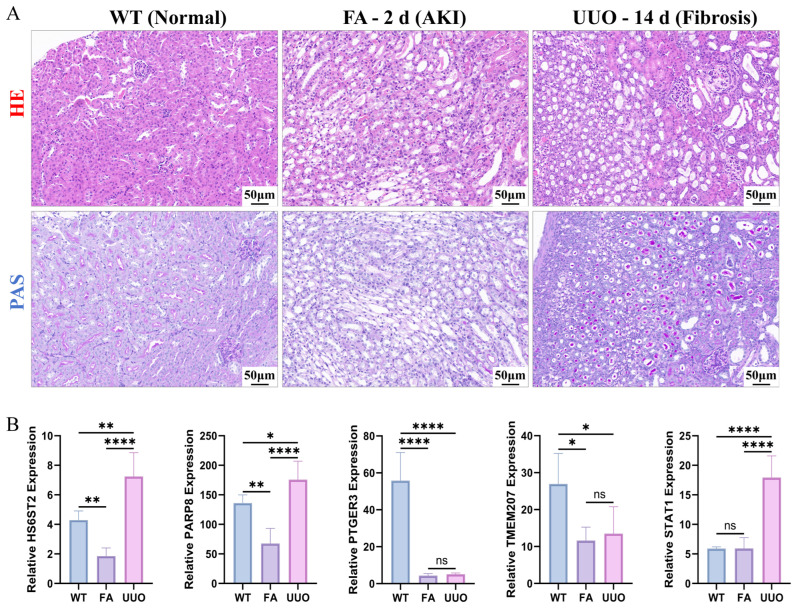
Histological examination and biomarker expression analysis in mouse renal tissues. (**A**) Representative images of hematoxylin and eosin (H&E) and periodic acid–Schiff (PAS) staining of kidney sections from the normal control group (WT), the folic acid-induced acute kidney injury group (FA, AKI), and the unilateral ureteral obstruction-induced chronic renal fibrosis group (UUO, Fibrosis). Staining was used to evaluate renal tubular and glomerular structure and to assess pathological injury. Scale bar: 50 μm; (**B**) mRNA expression levels of HS6ST2, PARP8, PTGER3, TMEM207, and STAT1 in kidney tissues of the WT, FA, and UUO groups (n = 3) were measured by qPCR and normalized to the reference gene Rps16 (ribosomal protein small subunit 16). Bar graphs show the relative expression of the five predicted biomarkers across the three groups. Data are presented as mean ± SD; ns, not significant (*p* > 0.05); * *p* < 0.05, ** *p* < 0.01, and **** *p* < 0.0001.

H&E staining revealed that the normal group exhibited intact renal architecture with well-aligned tubules and no significant inflammation. The FA-2 d group showed typical acute tubular injury, including epithelial vacuolization, necrosis, and interstitial inflammatory infiltration. In contrast, the UUO-14 d group displayed chronic fibrotic features such as tubular atrophy, interstitial expansion, and fibrous tissue deposition. PAS staining further delineated structural alterations in the tubular basement membrane (TBM) and brush border. In the normal group, the TBM was continuous, and the brush border remained intact. The FA-2 d group exhibited disruption of the TBM and loss of the brush border, while the UUO-14 d group presented with fragmented TBM, marked tubular atrophy, and increased extracellular matrix deposition in the expanded interstitium. In summary, these histopathological findings confirm that the FA and UUO models successfully recapitulate the characteristic features of acute tubular injury and chronic renal fibrosis, respectively, and clearly distinguish the different stages of renal pathology (Figure 7A).

qPCR analysis revealed differential expression of five core biomarkers in renal injury and fibrotic tissues compared with the normal control group (Figure 7B). Specifically, STAT1 and PARP8 were upregulated in disease models, whereas PTGER3 and TMEM207 were downregulated. Notably, HS6ST2 exhibited a temporospatial expression pattern: its expression was decreased in early acute kidney injury (FA model) but significantly increased during the chronic fibrotic phase (UUO model, 14 d). This expression profile aligned with the bioinformatics predictions. Previous temporal analysis indicated that HS6ST2 primarily functions in the CTAL, a segment susceptible to early damage. Consequently, HS6ST2 downregulation during the acute phase may reflect early CTAL damage, whereas its upregulation in chronic fibrosis may indicate compensatory and/or repair-associated responses. These findings are consistent with our earlier prediction that HS6ST2 expression is markedly elevated in the late stage of differentiation (Figure 5H). Collectively, the experimental results validate the biomarker expression patterns predicted by bioinformatics analysis.

### 3.7. The Biomarkers Exhibited Strong Binding Affinity with Their Targeted Drugs

**Figure 8:**
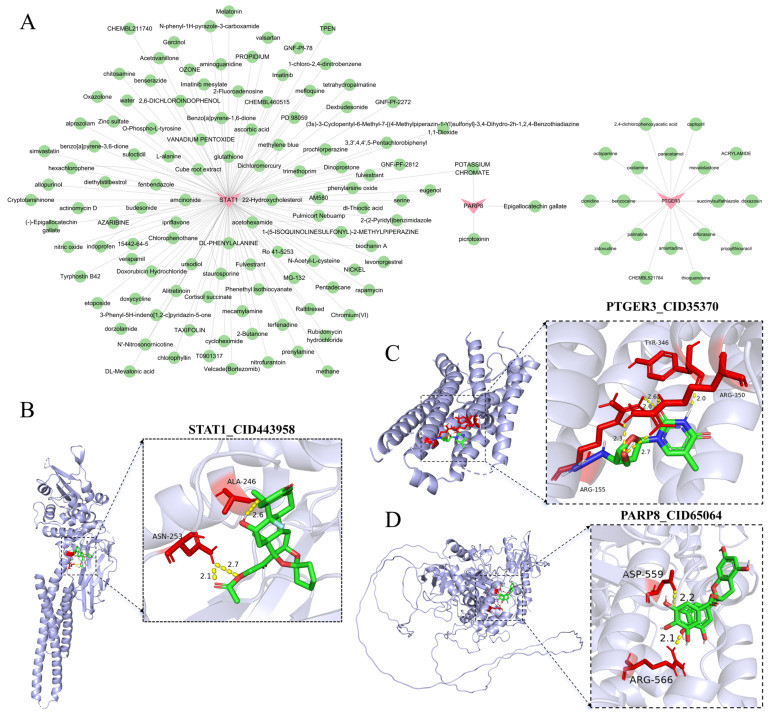
Drug prediction and molecular docking of biomarkers. (**A**) Drug–gene network of biomarkers. Drugs are colored green, and target proteins are colored pink. (**B**) Molecular docking between STAT1 and amcinonide. (**C**) Molecular docking between PTGER3 and zidovudine. (**D**) Molecular docking between PARP8 and epigallocatechin gallate.

Through DSigDB, 131 drugs targeted biomarkers were predicted. Among them, STAT1 was associated with 118 drugs, PARP8 with 3 drugs, and PTGER3 with 18 drugs. Unfortunately, there were no corresponding drugs for HS6ST2 and TMEM207 (Figure 8A). Amcinonide, zidovudine, and epigallocatechin gallate were selected for molecular docking based on their lowest *p*-values. The amino acid residues of STAT1 formed three hydrogen bonds with amcinonide, exhibiting a molecular binding energy of −7.13 kcal/mol (Figure 8B). PTGER3 established five hydrogen bonds with zidovudine, showing a binding energy of −6.64 kcal/mol (Figure 8C). PARP8 interacted with epigallocatechin gallate through two hydrogen bonds, with a binding energy of −7.57 kcal/mol (Figure 8D, Table 2).

## 4. Discussion

The pronounced cellular heterogeneity and complex intercellular communication within the renal fibrotic microenvironment have long constituted major obstacles to elucidating disease mechanisms and developing targeted therapeutic strategies [33,34]. Conventional bulk transcriptomics lacks the resolution to distinguish distinct cellular subtypes, whereas standalone single-cell analyses often fail to establish direct associations with clinical phenotypes. To overcome these limitations, this study innovatively integrated the Scissor algorithm with a customized CIBERSORTx signature matrix constructed from single-cell data. This strategy not only enabled high-resolution deconvolution of cell-type composition from bulk data but also allowed precise phenotypic association at the subpopulation level. Using this approach, we identified CTAL and aTAL cells as two key and functionally distinct TAL subtypes involved in RF progression, and further discovered five closely associated biomarkers: STAT1, PARP8, HS6ST2, PTGER3, and TMEM207.

The thick ascending limb plays a crucial role in maintaining water-electrolyte homeostasis and tubuloglomerular feedback, and its injury-induced cellular plasticity under chronic stress is considered a key driver of fibrosis [35,36,37]. Our findings indicate that CTAL is predominantly enriched in the early pseudotime stage of differentiation, with its proportion decreased and cell–cell communication significantly weakened in RF. This suggests that CTAL dysfunction may be an early event in RF, potentially initiating pathological processes by disrupting normal filtration feedback regulation [38,39,40,41]. In contrast, aTAL—a subtype emerging under chronic stress—is mainly present at intermediate pseudotime stages and shows a marked increase in proportion in RF. aTAL may represent a metabolically and functionally reprogrammed phenotype adopted by TAL cells to adapt to injury. Its sustained intercellular communication activity may reflect a compensatory attempt to preserve homeostasis; however, it may also inadvertently amplify profibrotic signaling [42].

At the molecular level, the five biomarkers form a network linked to TAL subtype functions. STAT1 (upregulated in aTAL) is a known pro-inflammatory and pro-fibrotic transcription factor whose activation exacerbates renal inflammation [43,44,45]. PARP8 (upregulated in aTAL), a member of the PARP family, is structurally related to PARP1, which has been shown to promote fibrosis through mediating inflammation, oxidative stress, and other pathways [46,47,48,49,50,51,52]. Conversely, HS6ST2, PTGER3, and TMEM207 (all downregulated in CTAL) are implicated in key physiological processes, including the maintenance of basement membrane integrity [53], anti-inflammatory and vasodilatory protection [54,55], and epithelial cell polarity/transport [56], respectively. Their collective downregulation likely compromises tubular structural integrity and defensive capacity.

Importantly, the expression patterns of these biomarkers were independently validated by qPCR in two well-established mouse models: FA–induced AKI and unilateral UUO–induced chronic renal fibrosis. The experimental results corroborated the bioinformatic predictions: STAT1 and PARP8 were significantly upregulated in disease groups; PTGER3 and TMEM207 were downregulated; and HS6ST2 exhibited a dynamic temporal profile, with initial downregulation during the acute phase followed by upregulation in the chronic phase, consistent with its predicted increase in late pseudotime (Figure 5H). Notably, as a member of the heparan sulfate-modifying enzyme family, HS6ST2 exhibits differential functions in acute versus chronic disease states. The literature indicates that, in acute inflammatory conditions such as osteoarthritis, HS6ST2 expression is downregulated and is associated with reduced chondrocyte activity [57,58]. Conversely, across chronic fibrotic models affecting the heart, liver, and kidney, HS6ST2 is selectively upregulated in activated fibroblasts, where it promotes extracellular matrix deposition, including collagen production, thereby driving fibrotic progression [59]. Consistent with these observations, HS6ST2 upregulation during the chronic phase of the UUO model corroborates prior reports and suggests that HS6ST2 may serve as a stage-specific biomarker. Furthermore, its dynamic expression may reflect the pathological transition from acute injury to chronic fibrotic remodeling, and its context-dependent roles—anti-inflammatory in acute settings and pro-fibrotic in chronic settings—require further mechanistic validation.

Gene Set Enrichment Analysis further revealed that these biomarkers are collectively associated with metabolic reprogramming pathways (e.g., oxidative phosphorylation, butanoate metabolism) and immune dysregulation pathways (e.g., graft-versus-host disease and lupus). This outlines a complex interplay between energy metabolism disturbances and immune-inflammatory responses during RF progression. Building on this molecular network, our drug prediction efforts identified several candidate compounds with high binding affinity, such as amcinonide (targeting STAT1), zidovudine (targeting PTGER3), and epigallocatechin gallate (targeting PARP8), offering new avenues for drug repurposing or combination therapy.

Several limitations warrant consideration, including reliance on public datasets that may introduce batch-related confounding and the need for additional functional experiments to elucidate the molecular mechanisms governing CTAL/aTAL subtype transitions and biomarker activity. Nevertheless, by integrating multi-omics computational analyses with in vivo experimental validation, this study systematically delineates the central contribution of TAL cellular heterogeneity to RF and identifies subtype-specific biomarkers and candidate therapeutic targets with translational relevance. Consequently, these findings provide a foundation for the future development of cell-resolved precision diagnostic and targeted therapeutic strategies for kidney disease.

## 5. Conclusions

In conclusion, this study systematically analyzed TAL cell heterogeneity under RF conditions and identified two principal subtypes (CTAL and aTAL) by integrating single-cell and bulk transcriptomic datasets. Based on this, five biomarkers (STAT1, PARP8, HS6ST2, PTGER3, TMEM207) closely associated with these subtypes were identified, and their enrichment patterns in metabolic reprogramming and immune dysregulation pathways were clarified. This provides a molecular mechanistic framework for elucidating the interactions among inflammation, metabolism, and fibrosis during RF progression. Future studies should validate these findings in independent cohorts and perform functional assays to elucidate the regulatory mechanisms of TAL subtype transitions and biomarker functions. These efforts are essential to advance the development of cell-targeted therapies for kidney disease.

## Figures and Tables

**Table 1:** RT-qPCR primers.

Gene	Forward Primer (5′-3′)	Reverse Primer (5′-3′)
*STAT1*	TCACAGTGGTTCGAGCTTCAG	GCAAACGAGACATCATAGGCA
*PARP8*	TAAATCGCACAAACTTTTGGGC	TCTCCAGAACAAGATCGAGTCAA
*HS6ST2*	ACCGGGGAAGTCAGAAGCA	CTCTACGCTCCCTATGTAGTCAT
*PTGER3*	CCGGAGCACTCTGCTGAAG	CCCCACTAAGTCGGTGAGC
*TMEM207*	TGCTCTCGGATCTATCCTGTG	ATTCCGCACCTTTTCAGCCA
*mRps16*	CGTGCTTGTGCTCGGAGCTA	GCTCCTTGCCCAGAAGCAAA

**Table 2:** Binding energy of biomarkers with targeted compounds.

Symbol	UniProt Accession	Molecule Name	CID	Affinity (kcal/mol)	Hydrogen Bonds
STAT1	P42224	Amcinonide	CID443958	−7.13	3
PTGER3	P43115	zidovudine	CID35370	−6.64	5
PARP8	Q8N3A8	Epigallocatechin Gallate	CID65064	−7.57	2

## Data Availability

The original contributions presented in this study are included in the article and Supplementary Materials. Further inquiries can be directed to the corresponding authors.

## Supplementary Materials

The following supporting information can be downloaded at: https://www.mdpi.com/article/10.3390/cimb48020215/s1.
