# Supplementary figures and images for: Scissor–CIBERSORTx Deconvolution Reveals Functional Heterogeneity of CTAL/aTAL Cells and Associated Biomarkers in Renal Fibrosis
Source: Curr Issues Mol Biol. 2026 Feb 16;48(2):215. doi: 10.3390/cimb48020215 (PMC12939945; doi:10.3390/cimb48020215)

## Supplementary Information

A

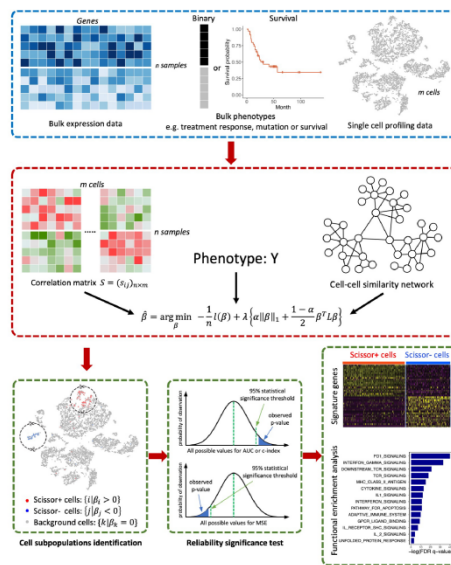

B

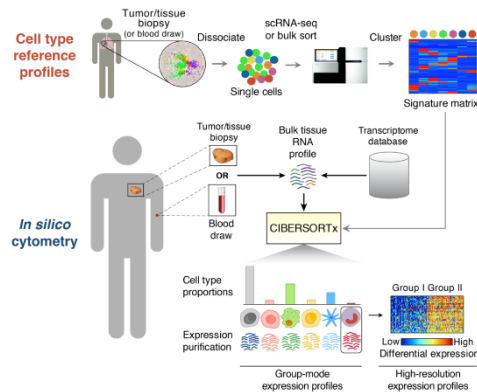

C

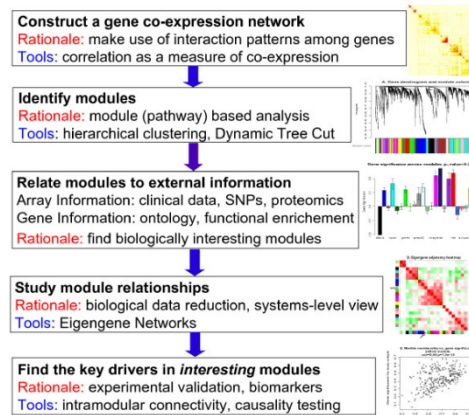

**Figure S1.** Workflow diagram of (A) Scissor (B) CIBERSORTx (C) WGCNA.

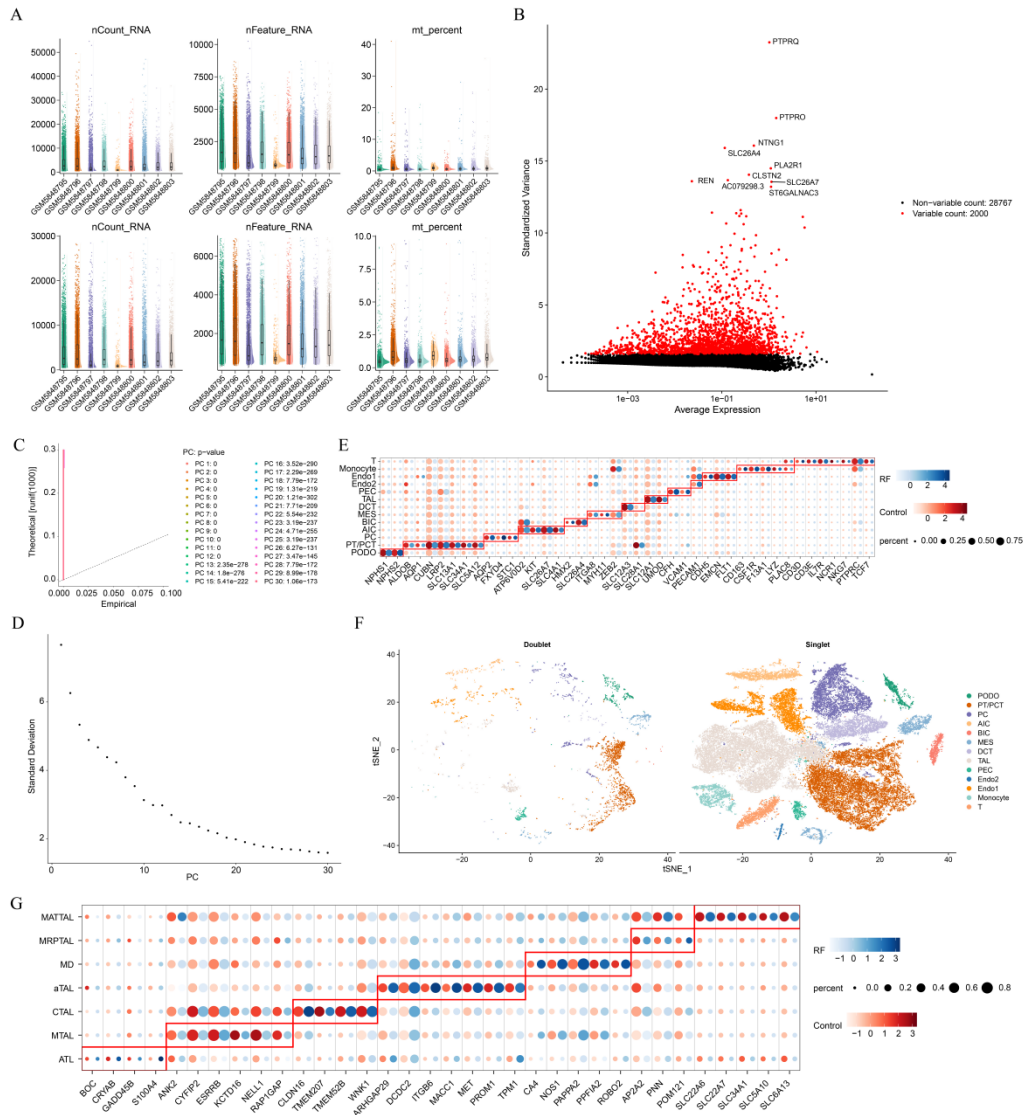

Supplement: Supplementary file 1 [file cimb-48-00215-s001.zip › Supplementary Information.pdf]
